# Increasing transparency and accountability in national pharmaceutical systems

**DOI:** 10.2471/BLT.17.206516

**Published:** 2018-08-30

**Authors:** Anne Paschke, Deirdre Dimancesco, Taryn Vian, Jillian C Kohler, Gilles Forte

**Affiliations:** a Department of Political Sciences, University of Hamburg, Max-Brauer-Allee 60, 22767 Hamburg, Germany.; b WHO Essential Medicines and Health Products Department, World Health Organization, Geneva, Switzerland.; c School of Public Health, Boston University, Boston, United States of America.; d Leslie Dan Faculty of Pharmacy, University of Toronto, Toronto, Canada.

## Abstract

Access to safe, effective, good-quality medicines can be compromised by poor pharmaceutical system governance. This system is particularly vulnerable to inefficiencies and to losses from corruption, because it involves a complex mix of actors with diverse responsibilities. A high level of transparency and accountability is critical for minimizing opportunities for fraud and leakage. In the past decade, the Good Governance for Medicines programme and the Medicines Transparency Alliance focused on improving accountability in the pharmaceutical system and on reducing its vulnerability to corruption by increasing transparency and encouraging participation by a range of stakeholders. Experience with these two programmes revealed that stakeholders interpreted transparency and accountability in a range of different ways. Moreover, programme implementation and progress assessments were complicated by a lack of clarity about what information should be disclosed by governments and about how greater transparency can strengthen accountability for access to medicines. This article provides a conceptual understanding of how transparency can facilitate accountability for better access to medicines. We identified three categories of information as prerequisites for accountability: (i) standards and commitments; (ii) decisions and results; and (iii) consequences and responsive actions. Examples are provided for each. Conceptual clarity and practical examples of the information needed to ensure accountability can help policy-makers identify the actions required to increase transparency and accountability in their pharmaceutical systems. We also discuss factors that can hinder or facilitate the use of information to hold to account those responsible for improving access to medicines.

## Résumé

L'accès à des médicaments sûrs, efficaces et de bonne qualité peut être compromis par la mauvaise gouvernance du secteur pharmaceutique. Celui-ci est particulièrement exposé aux inefficacités et aux pertes dues à la corruption, du fait qu'il implique un ensemble complexe d'acteurs aux différentes responsabilités. Une grande transparence et l'obligation de rendre des comptes sont indispensables pour minimiser les possibilités de fraude et de fuite. Durant la dernière décennie, le programme de Bonne gouvernance dans le secteur pharmaceutique et l'initiative Medicines Transparency Alliance se sont attachés à améliorer la reddition de comptes dans le secteur pharmaceutique et à réduire la vulnérabilité de ce dernier face à la corruption, en augmentant la transparence et en encourageant la participation de nombreuses parties prenantes. L'expérience de ces deux programmes a révélé que les parties prenantes interprétaient la transparence et la reddition de comptes de différentes manières. En outre, la mise en œuvre des programmes et l'évaluation des progrès ont été compliquées par un manque de clarté quant aux informations que les gouvernements devaient communiquer et à la manière dont une plus grande transparence pouvait renforcer la reddition de comptes et l'accès aux médicaments. Cet article entend expliquer en quoi la transparence peut faciliter la reddition de comptes et améliorer l'accès aux médicaments. Nous avons identifié trois catégories d'informations nécessaires à la reddition de comptes: (i) normes et engagements; (ii) décisions et résultats; (iii) conséquences et mesures réactives. Des exemples sont présentés pour chaque catégorie. Un cadre théorique clair et des exemples pratiques d’informations nécessaires pour assurer la reddition de comptes peuvent aider les responsables politiques à déterminer les actions requises pour augmenter la transparence et la reddition de comptes dans leurs systèmes pharmaceutiques. Nous évoquons aussi les facteurs qui peuvent entraver ou faciliter l'utilisation des informations et engager la responsabilité des personnes chargées d'améliorer l'accès aux médicaments.

## Resumen

El acceso a medicamentos seguros, eficaces y de buena calidad puede verse comprometido por una mala gestión del sistema farmacéutico. Este sistema es particularmente vulnerable a las ineficiencias y a las pérdidas derivadas de la corrupción porque implica una compleja mezcla de distintos participantes con diversas responsabilidades. Un alto nivel de transparencia y responsabilidad es fundamental para minimizar las oportunidades de fraude y filtraciones. En la última década, el programa de Buena Gestión de los Medicamentos y la Alianza para la Transparencia de los Medicamentos se han centrado en mejorar la responsabilidad del sistema farmacéutico y en reducir su vulnerabilidad a la corrupción mediante el aumento de la transparencia y el fomento de la participación de una serie de partes interesadas. La experiencia con estos dos programas reveló que las partes interesadas interpretaban la transparencia y la responsabilidad de diversas maneras. Además, la implementación de los programas y las evaluaciones de progreso se complicaron debido a la falta de claridad sobre la información que deben divulgar los gobiernos y sobre cómo una mayor transparencia puede fortalecer la responsabilidad en el acceso a los medicamentos. Este artículo ofrece una explicación conceptual de cómo la transparencia puede facilitar la responsabilidad para un mejor acceso a los medicamentos. Se han identificado tres categorías de información como requisitos previos a la responsabilidad: (i) normas y compromisos; (ii) decisiones y resultados; y (iii) consecuencias y medidas de respuesta. Se ofrecen ejemplos de todas. La claridad de conceptos y los ejemplos prácticos de la información necesaria para garantizar la responsabilidad pueden ayudar a los responsables políticos a identificar las medidas necesarias para aumentar la transparencia y la responsabilidad en sus sistemas farmacéuticos. También se analizan los factores que dificultan o facilitan el uso de la información para responsabilizar a las personas responsables de mejorar el acceso a los medicamentos.

## ملخص

إن إمكانية الحصول على أدوية آمنة وفعالة وذات نوعية جيدة، يمكن أن تتأثر بسوء إدارة أنظمة الدوائية. يكون هذا النظام على وجه التحديد عرضة لعدم الكفاءة والخسائر الناجمة عن الفساد لأنه ينطوي على مزيج معقد من الجهات الفاعلة ذات المسؤوليات المتنوعة. ويُعد توافر مستوى عال من الشفافية والمساءلة أمراً بالغ الأهمية للحد من فرص الاحتيال والتسرب. في العقد الماضي، ركز كل من برنامج الحكم الرشيد للأدوية، وتحالف الشفافية في الأدوية، على تحسين المساءلة في النظام الدوائي، والحد من تعرضه للفساد من خلال زيادة الشفافية وتشجيع المشاركة بواسطة قطاع من الجهات المعنية. وكشفت التجربة مع خلال هذين البرنامجين أن الجهات المعنية قد فسرت الشفافية والمساءلة عبر عدة طرق مختلفة. وعلاوة على ذلك، فإن تنفيذ البرامج وكذلك عمليات تقييمات التقدم، كانت معقدة بسبب الافتقار إلى الوضوح بشأن المعلومات التي ينبغي أن تفصح عنها الحكومات، وعن كيف يمكن لزيادة الشفافية أن تعزز المساءلة فيما يتعلق بالحصول على الأدوية. تقدم هذه المقالة فهماً مفاهيمياً لقدرة الشفافية على تسهيل المساءلة فيما يتعلق بالحصول على الأدوية بشكل أفضل. لقد حددنا ثلاث فئات من المعلومات كمتطلبات مسبقة للمساءلة: (1) المعايير والالتزامات؛ و(2) القرارات والنتائج؛ و(3) النتائج وإجراءات الاستجابة. ويتم توفير أمثلة لكل منها. يمكن للوضوح المفاهيمي، والأمثلة العملية للمعلومات المطلوبة لضمان المساءلة، أن تساعد واضعي السياسات في تحديد الإجراءات المطلوبة لزيادة الشفافية والمساءلة في نظمهم الدوائية. كما أننا نناقش العوامل التي يمكن أن تؤدي إلى تعويق أو تسهيل استخدام المعلومات لمحاسبة المسؤولين بخصوص تحسين الحصول على الأدوية.

## 摘要

药物系统治理不善会影响安全、有效、优质药品的获取得不到保障。由于其涉及承担不同责任行为者的复杂组合,该系统尤其容易受到效率低下、因腐败带来损失的影响。为了最大限度降低欺诈和腐败的几率,高水平的透明度和问责制至关重要。在过去十年中,药物良好管理规划 (Good Governance for Medicines Programme) 和药品透明度联盟 (Medicines Transparency Alliance) 的关注重点在于改善药物系统的问责制,并通过提升药物系统的透明度,鼓励一系列利益相关者的参与,从而降低药物系统的腐败程度。这两个项目的经验表明,利益相关者以各种不同的方式阐释透明度和问责制。此外,由于并未明确政府应披露哪些信息以及如何提升透明度来完善药品获取的问责制,项目实施和进展评估变得复杂。本文提供了一种概念性理解,阐明了透明度如何促进问责制,以更佳途径获取药品。我们确定了三类信息作为问责制的先决条件:(i) 标准与承诺;(ii) 决策与结果;(iii) 后果与应对措施。并为每项提供了示例。确保问责制所需信息的概念清晰、示例可行能帮助政策制定者确定所需行动,以提高其药物系统透明度、健全相关问责制。我们还讨论了可能阻碍或促进信息使用的因素,以便对改善药品获取途径的责任人进行问责。

## Резюме

Доступ к безопасным, эффективным, качественным лекарственным препаратам может быть сведен на нет плохой организацией управления в фармацевтическом секторе. Эта система особенно уязвима для неэффективности и потерь, связанных с коррупцией, так как ей присуща сложная структура участников с различными обязанностями. Для сведения к минимуму мошеннических сделок и утечек средств крайне важно обеспечить высокий уровень прозрачности и подотчетности системы. В последнее десятилетние программа «Надлежащее управление в области лекарственных средств» (Good Governance for Medicines) и Альянс за прозрачность лекарственного обеспечения (Medicines Transparency Alliance) уделяют основное внимание подотчетности фармацевтических систем и снижению их уязвимости для коррупции за счет повышения прозрачности и привлечения целого ряда партнеров к участию в их работе. Опыт этих двух программ показал, что партнеры весьма по-разному интерпретируют понятия прозрачности и подотчетности. Более того, выполнение программы и оценка хода ее осуществления осложнялись отсутствием ясности в вопросе того, какую информацию должны раскрывать правительства стран и как именно повышение прозрачности сделок может укрепить подотчетность для доступа к лекарственным препаратам. Данная статья обеспечивает концептуальное понимание того, каким образом прозрачность действий может способствовать подотчетности для упрощения доступа к лекарственным препаратам. Авторы выделили три категории информации в качестве необходимых условий подотчетности: (i) стандарты и обязательства; (ii) решения и результаты; (iii) последствия и ответные действия. Для каждой категории приводятся примеры. Концептуальная ясность и практические примеры информации, необходимой для обеспечения подотчетности, помогут лицам, принимающим стратегические решения, определиться с мерами, необходимыми для повышения прозрачности и подотчетности соответствующих фармацевтических систем. Авторы также обсуждают факторы, которые могут содействовать или препятствовать использованию информации для привлечения к ответственности лиц, в чьи обязанности входит улучшение доступа к лекарственным средствам.

## Introduction

The sustainable development goals (SDGs) highlight the critical role of governance across all sectors, including health.^1^ SDG 16 concerns: (i) promoting the rule of law; (ii) preventing corrupt practices; (iii) developing accountable and transparent institutions; (iv) ensuring responsive, inclusive and participatory decision-making processes; and (v) ensuring public access to information. In addition, SDG 3 incorporates the target of achieving universal health coverage (UHC), including access for all to safe, effective, good-quality and affordable essential medicines and vaccines.^2^ Robust public accountability and participation mechanisms are widely accepted to be indispensable for pursuing UHC.^3^ At least half the world’s population still lacks access to good-quality essential services, such as those providing medicines to protect and promote health.^4^ In many parts of the world, out-of-pocket health expenditure remains high and an estimated 800 million people spend more than 10% of their household budget on health care.^4^^–^^6^ Since medicines often make up a large portion of health spending, improving access to medicines is central to achieving UHC.

**Fig. 1:**
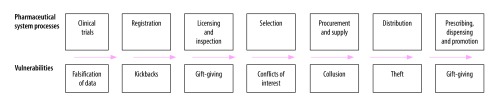
Potential vulnerabilities in the pharmaceutical system

The 2010 World Health Report stated that an estimated 20–40% of potential health gains from health spending are lost through inefficiencies, such as: (i) the underuse of generic medicines and the overuse of overpriced medicines;^7^ (ii) the availability of substandard and falsified medical products; (iii) inappropriate and ineffective prescribing; and (iv) losses from the health system due to waste, corruption and fraud.^8^^,^^9^ In the absence of good governance, the pharmaceutical system, with its numerous stakeholders and complex networks, is particularly vulnerable to such inefficiencies, particularly to losses from corruption.^10^^,^^11^ Weak institutional capacity for implementing guidelines and standard operating procedures contributes to inadequate performance. Moreover, corruption, which is defined by Transparency International as “the abuse of entrusted power for private gain,”^12^ reduces the resources available for medicines: it lowers the government’s capacity to provide good-quality essential medicines and contributes to medicines being out of stock or inaccessible. Fig. 1 shows examples of vulnerabilities in the pharmaceutical system.^13^

## Transparency and accountability

Better access to safe, effective, good-quality medicines requires stronger governance of the pharmaceutical system and transparency and accountability have consistently been identified as key elements of governance.^14^ Transparency and accountability can reduce vulnerability to corruption and unethical practices and improve public trust in government institutions.^15^^,^^16^ In recent years, new standards for transparency in the pharmaceutical system have emerged. A major advance was the call for the public disclosure of clinical trial results.^17^ Another was the demand for transparency on medicine production costs to provide a basis for fair pricing.^18^ In addition, new mechanisms for ensuring transparency about industry’s payments to physicians have been established; for example, recent legislation in Ontario, Canada, which was modelled on the United States' Physician Payments Sunshine Act.^19^

Frameworks for conceptualizing or assessing governance in the health and pharmaceutical sectors often include transparency and accountability and have been developed following extensive consultations with multiple stakeholders.^20^^–^^28^ Yet, despite growing interest, little has been published on practical approaches to improving transparency and accountability in the pharmaceutical system or on evidence of their effects on health system outcomes. In particular, it is unclear what information should be made transparent and how that information should be used. Another obstacle is that transparency and accountability are often defined as distinct concepts. We see them as actually being closely related and believe that an understanding of how transparency can increase accountability is crucial for identifying the types of information that should be made publicly available.

Here we propose an approach to identifying key information across the pharmaceutical system that can help policy-makers and other stakeholders strengthen accountability and we illustrate its application. In developing this approach, we have drawn on the experience of two international programmes that focused on transparency, accountability and anticorruption practices in the pharmaceutical system.

## Learning from practice

Recently, two global initiatives sought to improve transparency and accountability in the pharmaceutical system: the Medicines Transparency Alliance and the Good Governance for Medicines programme led by the World Health Organization (WHO). The Medicines Transparency Alliance, which operated from 2008 to 2015, had the objective of improving access to medicines through enhanced transparency and accountability in the pharmaceutical system and adopted a multistakeholder approach.^29^^,^^30^ However, different conceptual interpretations in participating countries led to varied approaches to implementation, which complicated the assessment of transparency and accountability.^16^ Nevertheless, a cross-case analysis found strong evidence that the Medicines Transparency Alliance resulted in greater transparency in participating countries.^16^^,^^31^ Whether or not activities such as policy dialogue between stakeholders and capacity building in civil society will help achieve greater government accountability depends on how well these activities are sustained over time.^16^^,^^31^ The WHO Good Governance for Medicines programme, which has been operational since 2004, seeks to address vulnerability to corruption and to strengthen health systems. The programme involves: (i) the assessment of transparency; (ii) the development of a national governance framework for the pharmaceutical sector; and (iii) implementation of this national framework. The first version of the programme’s assessment tool has been used in 38 countries.^32^ An evaluation of the Good Governance for Medicines programme in 2012 confirmed that it had been instrumental in increasing awareness of transparency and good governance in the pharmaceutical system, both globally and in individual countries, and that it had increased transparency through the publication of previously unavailable pharmaceutical information.^33^

## Developing a new approach

After 10 years of experience with the Good Governance for Medicines programme, WHO recognized that improvements were needed to simplify the assessment method and to clearly define which aspects of governance should be assessed.^32^^,^^34^ With the aim of revising the transparency assessment instrument, several meetings were held to collect the views of a range of stakeholders, such as: (i) national assessors who were responsible for conducting transparency assessments; (ii) representatives of countries participating in the Medicines Transparency Alliance and the Good Governance for Medicines programme; (iii) governance and transparency experts from academia; and (iv) representatives of international organizations and of national and international civil society organizations, participants are listed in the referenced meeting reports.^34^^–^^37^ We also carried out an online search of the literature using combinations of the terms *pharmaceutical system*, *pharmaceutical sector*, *health system*, *transparency*, *accountability*, *participation*, *governance* and *good governance*. Experts in the field were consulted to identify additional relevant literature. In addition, we reviewed key pieces of documentation, such as WHO’s National Regulatory Authority Assessment tool,^24^ *Managing Access to Medicines and Health Technologies* by Management Sciences for Health,^25^ and all relevant WHO pharmaceutical guidelines.

The reviews of the Medicines Transparency Alliance and the Good Governance for Medicines programme and the assessment instrument revision process revealed that there was no uniform conceptualization or understanding of transparency or accountability either across countries or among stakeholders within individual countries.^31^^,^^33^ In many countries, it was unclear what transparency and accountability meant and how they could be achieved. In particular, there was a lack of practical approaches for identifying information that should be made public and for demonstrating how such information could be used to strengthen accountability. Consequently, we felt it was essential to illustrate the relationship between transparency and accountability as this would help countries identify the key information that should be made transparent and to show how it could be used to foster government accountability. The lessons learnt from the two global initiatives, the literature search and the consultations informed the development of the conceptual approach presented here. We hope this approach will help the development of practical measures for improving transparency and accountability in the pharmaceutical system and enable better monitoring of the measures implemented.

## Conceptual background

**Fig. 2:**
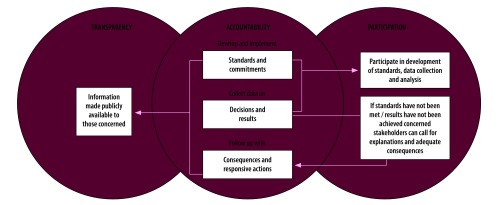
Facilitation of accountability through transparency and participation in the pharmaceutical system

Note: We divided the types of information regarded as a prerequisite for accountability into three categories: (i) standards and commitments; (ii) decisions and results; and (iii) consequences and responsive actions.

We identified a specific understanding of the concepts of transparency and accountability that is useful for operationalizing their application to the pharmaceutical system. Transparency is both an end in itself and is instrumental in enabling accountability. On the one hand, transparency has an intrinsic value as a normative principle that guides well-governed, democratic systems. On the other hand, it is a necessary, but not a sufficient condition for accountability.^38^ One current theory of change suggests that transparency informs stakeholders and, thereby, facilitates their participation in policy processes, which enables accountability.^39^ At the core of accountability is the process of being called to account for one’s actions through external scrutiny.^40^ Hence, when institutions make information publicly available, stakeholders can use this information to call those responsible in institutions to account, this may involve sanctions, compensation or remediation, should standards or commitments not have been met.^41^ We, therefore, understand accountability to mean that individuals and institutions: (i) are responsible for acting according to certain standards and commitments; (ii) are answerable for their actions; and (iii) will face consequences when standards or commitments are not met. Transparency is understood as governments making information publicly available so that their actions and decisions are visible and understandable to the public and so they can, therefore, be held to account. Fig. 2 illustrates how transparency and participation can enable accountability in the pharmaceutical system.

## Information required for accountability

On the basis of the review of the two global initiatives and the revision process, we identified key information about the pharmaceutical system that should be in the public domain. We regard this information as a prerequisite for accountability and divide it into three categories: (i) standards and commitments; (ii) decisions and results; and (iii) consequences and responsive actions. The aim was to help stakeholders understand the different types of information that, when in the public domain, can be used to establish accountability. A full list of the information we identified has been incorporated into a revised transparency assessment tool using the approach described here and has been field tested by WHO in the Netherlands, which served as an example of a centralized pharmaceutical system, and in the province of Ontario, Canada, which served as an example of a decentralized pharmaceutical system. Subsequently, the approach was field tested by local assessors in Fiji, Malawi, Malaysia, Mongolia and Serbia.^37^

### Standards and commitments

Making standards and commitments public enables stakeholders to understand who is accountable for what. In the pharmaceutical system, standards and commitments are manifested in different forms. For example, pharmaceutical laws and regulations provide the legal standards for ensuring that the manufacture, trade and use of medicines are consistent with the delivery of safe and effective products. In turn, guidelines and procedures set the standards according to which regulations should be implemented. National medicines policies demonstrate commitments to goals and provide a guide for action, which may include priorities for medium- and long-term goals and the main strategies for attaining them. The budget required for attaining goals represents the financial commitment the government must make to achieve them. In addition, an essential medicines list can also be considered as a commitment to making medicines available. Examples of standards and commitments affecting the pharmaceutical system are listed in Box 1.

Box 1 **Examples of pharmaceutical system standards and commitments that should be publicly available**

• Legislation on freedom of information that defines what information should be made available to the public;

• Regulation or policy to protect whistle-blowers;

• Policy to manage conflicts of interest among public employees;

• Standard operating procedures for inspectors;

• Requirements for the registration of clinical trials;

• Criteria for the recruitment of medicines selection committee members; and

• Requirements for dividing key procurement functions and responsibilities between different offices, committees or individuals

### Decisions and results

Disclosing decisions and results enables stakeholders to know whether or not agreed standards and commitments have been met. In particular, the publication of government decisions, meeting records, audit reports and monitoring and evaluation data provide the information needed to scrutinize whether or not the results achieved (e.g. in the form of process, output or outcome indicators) are consistent with standards and commitments. For example, technical evaluation reports on approved pharmaceutical products can be used to establish whether or not data have been submitted and appraised according to the standards stipulated by guidelines and procedures for marketing authorization. Box 2 provides additional examples of the information on government decisions and their results that should be publically available.

Box 2 **Examples of information that should be publically available on government decisions regarding the pharmaceutical system and on the results achieved**

• Indicators of medicines availability (e.g. facility-level stock-outs);

• Progress reports on the implementation of national medicines policies;

• A list or database of all pharmaceutical products registered in the country that is updated at least annually;

• A central register of payments made by pharmaceutical companies to health professionals and health-care organizations that lists both payers and recipients;

• A list of the names and roles of individuals appointed to tender committees;

• A list of contracts for publicly procured medicines, including those exempted from tendering; and

• A summary of the findings of warehouse audit reports

### Consequences and responsive actions

The final prerequisite for establishing accountability is the provision of information that enables stakeholders to understand the consequences of any deviations from agreed standards and commitments and to be aware of the responsive actions taken by government when these deviations occur. Actions may include requiring an individual or institution to address the issue for which they had been called to account by accepting sanctions, making redress or implementing a standard they failed to meet. Moreover, publication of the responsive actions taken by government demonstrates to stakeholders that public institutions are accountable. The consequences of deviations from standards may also include the revision of policies or laws, legal or juridical actions, or changes in resource allocation. Specific examples of the information that should be publically available on the consequences of deviating from standards are provided in Box 3.

Box 3 **Examples of information that should be publically available on the consequences of deviating from pharmaceutical system standards and on the responsive actions taken**

• Reports of how conflicts of interest were mitigated or resolved;

• A list of pharmaceutical establishments that had their licences revoked;

• Copies of warning letters demanding retractions or corrections that have been sent to pharmaceutical companies;

• Reports of research misconduct and associated corrective actions; and

• A list of suppliers suspended for not respecting their contracts

## Pharmaceutical system functions

Distinguishing different functions within the pharmaceutical system is useful when considering these three categories of information: we subdivided these functions into three cross-cutting functions and eight specific pharmaceutical system functions (Table 1). The three cross-cutting functions were identified during revision of the Good Governance for Medicines programme. It was found that information on medicines policies, codes of conduct and conflicts of interest was essential for ensuring transparency and accountability across all pharmaceutical system functions and should, therefore, receive more attention. The eight pharmaceutical system functions were based on the key functions assessed by the first version of the Good Governance for Medicines transparency assessment instrument.^32^

**Table 1:** Pharmaceutical system information for establishing accountability, by pharmaceutical system function in Canada

Pharmaceutical system function	Category of information that should be publicly available^a^		
	Standards and commitments	Decisions and results	Consequences and responsive actions
**Cross-cutting functions**			
Medicines policy	National medicines policy (see the 2006 *National pharmaceuticals strategy: progress report*)	Progress reports on the implementation of national medicines policy (the 2006 *National pharmaceuticals strategy: progress report*)	NA
Codes of conduct and anticorruption measures	Law or policy to protect whistle-blowers (the *Public servants disclosure protection act*)	Reports on the monitoring and evaluation of complaints and wrongdoing (*Complaints 2008–2015*)	NA
Conflicts of interest	Policy to manage conflicts of interest among experts serving as consultants or expert advisors to a national regulatory authority (the *Health Canada policy on external advisory bodies [2011]*)	A register, or registries, of declarations of the interests of members of pharmaceutical system committees	Descriptions of how conflicts of interest were mitigated or resolved: (case summaries and political activity reports from the Office of the Conflict of Interest Commissioner of Ontario)
**Specific pharmaceutical system functions**			
Registration and marketing authorization	Guidelines and procedures for marketing authorization (the guidance document *Preparation of drug regulatory activities in the common technical document [CTD] format*)	Technical evaluation reports and summaries of reports on approved products (product monographs in the drug product database)	Regulatory actions taken after marketing authorization approval (regulatory decision summaries for drugs)
Licensing	Official procedures for revoking the licence of any establishment that brought substandard or falsified medical products into the regulated supply chain (the *Policy on counterfeit health products*)	A list or database of pharmaceutical establishments with a licence or operating permit (the Ontario College of Pharmacists database of pharmacies and pharmacy professionals)	A list of pharmaceutical establishments that had their licences revoked or were closed down (lists of drug and health product inspections of drug manufacturers and wholesalers)
Inspections	Official rules prohibiting the operator of an inspected facility from directly paying for, or organizing, the travel, accommodation or catering for inspectors, except for fees paid to the medicines regulatory authority (the *Code of conduct: Canadian Food Inspection Agency [manufacturers/wholesalers/CRCs]*)	Summary findings of audit reports of inspectorates (drug programme activity reports from the Ontario Ministry of Health and Long-Term Care)	Corrective actions taken based on inspection results (the *Inspection Tracker: Drug Manufacturing Establishments* covering manufacturers and wholesalers)
Pharmaceutical promotion and independent information	Mandate for the body, or bodies, responsible for the active monitoring of promotional material (the guidance document *Health Canada and advertising preclearance agencies' roles related to health product advertising*)	Evaluation reports of sponsored medical educational events for health professionals (reports by the College of Physicians And Surgeons of Ontario)	Copies of warning letters demanding retractions or corrections sent to pharmaceutical companies (advisories, warnings and recalls)
Clinical trials oversight	Legal mandate for a body responsible for responding to allegations of ethical misconduct in clinical trials (the *Research Ethics Board – food and drug regulations*)	Decisions on applications for clinical trials (Health Canada’s clinical trials database)	Information about research misconduct and corresponding corrective actions (the clinical trial inspections database)
Medicines selection and reimbursement lists	Criteria for recruiting medicines selection committee members (the Canadian Drug Expert Committee terms of reference)	Statements made by public bodies, nongovernmental organizations and other interested parties about applications to, or decisions made by, the medicines selection committee (the Canadian Agency for Drugs and Technologies in Health’s Common Drug Review Patient Input system)	Details of the responses of the medicines selection committee to requests for clarification of decisions or the reinstatement of previously deleted or rejected medicines (the Canadian Agency for Drugs and Technologies in Health’s Common Drug Review reports)
Public procurement	Terms of reference for a tenders board or procurement committee or committees responsible for final contract decisions or adjudication (the terms of reference for the Committee to Evaluate Drugs)	List of contracts for publicly procured medicines exempted from tendering (the Ontario Ministry of Health and Long-Term Care’s *e-Formulary* under the Drugs Funded by Ontario Drug Benefit [ODB] programme)	Documents detailing the corrective measures that were enforced following a financial audit (the Ontario Ministry of Health and Long-Term Care’s drug programmes activity reports)
Distribution	Information system for monitoring the performance of, and evaluating, the distribution system (*National prescription drug utilization information system metadata*)	Information on confirmed drug seizures and alerts on substandard or falsified medical products (MedEffect Canada)	List of suppliers that were suspended for not having respected their contracts (executive officer lists of terminated health network system accounts)

NA: not available.

^a^ Details of the documents and information on the Canadian examples mentioned in the table are available from the corresponding author on request. These details were obtained when the revised Good Governance for Medicines programme’s transparency assessment tool was evaluated in the District of Ontario.

**Fig. 3:**
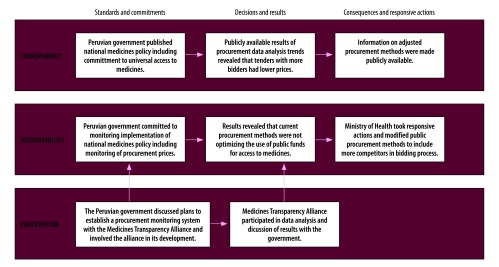
Influence of transparency and participation on accountability in the Peruvian pharmaceutical system, 2008–2015

Note: We divided the types of information regarded as a prerequisite for accountability into three categories: (i) standards and commitments; (ii) decisions and results; and (iii) consequences and responsive actions.

The matrix in Table 1 shows how the three categories can be used to identify, organize and assess the public availability of different types of information. Application of this matrix in the District of Ontario; Canada, helped identify information that was in the public domain. For example, the Government of Ontario has published guidelines on marketing authorization and registration and on technical evaluation reports for all pharmaceutical products. Publication enables stakeholders to determine whether registered products fulfil guideline criteria for registration. In addition, open information on the regulatory actions taken after marketing authorization makes it possible for the public to scrutinize whether adequate responsive actions were taken when registration standards were not met. Box 4 describes a country case study in Peru and, in Fig. 3, the application of our approach illustrates how the Peruvian government demonstrated accountability by making information about procurement prices publicly available and by taking responsive actions to decrease them.

Box 4 **Increasing transparency and accountability in the pharmaceutical system in Peru, 2008–2015**

The Medicines Transparency Alliance project in Peru, which began in 2008, supported the health ministry in monitoring and analysing national trends in pharmaceutical procurement. The analysis found that tenders with more bidders had lower prices. This finding and other evidence prompted national and regional public purchasers to take responsive actions, such as modifying their procurement methods to include more competitors (Fig. 3).^42^ In addition, the Medicines Transparency Alliance supported the Peruvian government in establishing a medicines price observatory as a mechanism for ensuring transparency on medicine prices, with real-time data being made available on the health ministry’s website. Furthermore, the government obliged all public and private medicine sellers in Peru to publish their retail prices through the medicines price observatory.^43^ Public, private and civil society organizations all participated in the analysis of data.^44^ These data have also been used to inform the government’s Inclusive Pharmacy project, which was developed as a responsive action to increase access among people with a low income. These examples demonstrate how publishing information can make governments more accountable to the public: in this case, the government’s commitment to increasing access to medicines can be evaluated against the measures it has taken to invest public procurement money responsibly, to promote the transparency of medicine prices and to increase affordability for patients. One remaining challenge is to sustain this commitment over time, for example, by continuing to hold multistakeholder group meetings to analyse data and by ensuring the medicines price observatory web site is constantly updated.

## Factors affecting transparency

Lessons learnt from both the Medicines Transparency Alliance and the Good Governance for Medicines programme illuminated several factors that can hinder or facilitate transparency and accountability. First, poor-quality information, or an overload of undigested data, may create confusion, rather than increase understanding and can actually hinder accountability. Governments should publish information in ways that allow concerned stakeholders to understand, analyse and use it to enhance accountability.^45^ Our approach to improving transparency does not assess whether each piece of documentation is produced in a timely manner or whether it is easy to understand. Instead, we chose to include timeliness as an assessment criterion only where we deemed it to be of particular importance: for example, lists and databases of pharmaceutical products registered in a country should be updated at least annually.

Second, the Medicines Transparency Alliance’s experience showed that, when civil society lacks the capacity to use information effectively or to engage in policy dialogue, its ability to hold government to account is weakened.^16^ Consequently, the publication of information should be complemented by efforts to build civil society capacity.^16^^,^^31^ Given the capacity to raise issues in public hearings or to publish open letters, civil society can put pressure on public institutions and demand justifications and responsive actions.

Another hindrance to transparency is that private entities, such as pharmaceutical companies, and civil society organizations often have different interests. For example, efforts made to foster transparency in the Good Governance for Medicines programme were sometimes met with resistance by interested parties that might have been exposed by greater transparency. In our understanding, the government is principally accountable to the public and should aim to achieve results in the public interest. Consequently, governments should focus on providing opportunities for public stakeholders (e.g. civil society) to participate in the development of standards and commitments and in monitoring decisions and results. Transparency about vested interests is especially important where the private sector is involved in implementing medicines policies. This is why we believe it is important to establish publicly accessible central registries of payments made by industry to health professionals and health-care organizations (Box 2). A critical facilitating factor is political support for transparency and accountability: accountability is difficult to achieve in the absence of the political will to disclose information and enable public participation.

Although the transparency assessment tool developed in the Good Governance for Medicines programme covers many pharmaceutical system functions, there remain areas that have not yet been addressed, but which are becoming the focus of global debate around transparency and medicines. These areas include calls to disclose the prices paid for publicly procured medicines, the cost of research and development, pharmaceutical production costs and profit margins.^46^

In conclusion, relevant, accessible and clear public information on the pharmaceutical system can be used to hold those responsible for providing medicines to account and can result in responsive actions that increase access. Our proposed division of pharmaceutical system information into three categories of information necessary for accountability is intended to help stakeholders recognize how transparency is essential for accountability in practice. This recognition could encourage public pharmaceutical institutions to regularly disclose key information about standards, decisions and results, and the consequences of deviating from standards and commitments. When coupled to mechanisms for public participation and for strengthening the capacity of civil society, the systematic disclosure of information can encourage its use and help improve accountability in the pharmaceutical system.

## References

[1] Resolution A/RES/70/1. Transforming our world: the 2030 agenda for sustainable development. In: Seventieth United Nations General Assembly, New York, 25 September 2015. New York: United Nations; 2015. Available from: http://www.un.org/ga/search/view_doc.asp?symbol=A/RES/70/1&Lang=E [cited 2018 Aug 27].

[2] Sustainable Development Goal 3: ensure healthy lives and promote well-being for all at all ages. New York: United Nations; 2018. Available from: http://www.un.org/sustainabledevelopment/health/ [cited 2017 Aug 19].

[3] Making fair choices on the path to universal health coverage. Final report of the WHO Consultative Group on Equity and Universal Health Coverage. Geneva: World Health Organization; 2014. Available from: http://www.who.int/choice/documents/making_fair_choices/en/ [cited 2017 Dec 10].

[4] Tracking universal health coverage: 2017 Global Monitoring Report. Geneva and Washington, DC: World Health Organization and The World Bank; 2017. Available from: http://www.who.int/healthinfo/universal_health_coverage/report/2017/en/ [cited 2017 Dec 10].

[5] Leive A, Xu K. Coping with out-of-pocket health payments: empirical evidence from 15 African countries. Bull World Health Organ. 2008 11;86(11):849–56. 10.2471/BLT.07.049403 19030690 PMC2649544

[6] Akazili J, McIntyre D, Kanmiki EW, Gyapong J, Oduro A, Sankoh O, et al. Assessing the catastrophic effects of out-of-pocket healthcare payments prior to the uptake of a nationwide health insurance scheme in Ghana. Glob Health Action. 2017;10(1):1289735. 10.1080/16549716.2017.1289735 28485675 PMC5496048

[7] Health systems financing: the path to universal coverage. The World Health Report 2010. Geneva: World Health Organization; 2010. Available from: http://www.who.int/whr/2010/en/ [cited 2017 Sep 8]. 10.2471/BLT.10.078741 PMC2878164 20539847

[8] Vian T. Review of corruption in the health sector: theory, methods and interventions. Health Policy Plan. 2008 3;23(2):83–94. 10.1093/heapol/czm048 18281310

[9] Cohen-Kohler J. Pharmaceuticals and corruption: a risk assessment. In: Kotalik J, Rodriguez D, editors. Global corruption report 2006. Transparency International. London: Pluto Press; 2006 pp. 77–85. Available from: http://www1.worldbank.org/publicsector/anticorrupt/corecourse2007/Pharmaceuticals.pdf [cited 2017 Jul 19].

[10] Kotalik J, Rodriguez D, editors. Global corruption report 2006. Transparency International. London: Pluto Press; 2006 [cited 2017 Nov 18]. Available from: Available from https://www.transparency.org/whatwedo/publication/global_corruption_report_2006_corruption_and_health

[11] MDG Gap Task Force report 2015: taking stock of the global partnership for development. New York: United Nations; 2015. Available from: https://www.un.org/development/desa/publications/mdg-gap-task-force-report-2015-taking-stock-of-the-global-partnership-for-development.html [cited 2018 Jan 8].

[12] What is corruption? How do you define corruption? [internet]. Berlin: Transparency International; 2018. Available from: https://www.transparency.org/what-is-corruption/#define [cited 2017 Sep 13].

[13] Cohen J, Mrazek M, Hawkins L. Corruption and pharmaceuticals: strengthening good governance to improve access. In: Campos JE, Pradhan S, editors. The many faces of corruption: tracking vulnerabilities at the sector level. Washington, DC: The World Bank; 2007. pp. 29–62. Available from: https://www.researchgate.net/publication/265066655_Corruption_and_Pharmaceuticals_Strengthening_Good_Governance_to_Improve_Access [cited 2018 Aug 27].

[14] Kohler JC, Mackey TK, Ovtcharenko N. Why the MDGs need good governance in pharmaceutical systems to promote global health. BMC Public Health. 2014 1 21;14(1):63. 10.1186/1471-2458-14-63 24447600 PMC3909282

[15] Barbazza E, Tello JE. A review of health governance: definitions, dimensions and tools to govern. Health Policy. 2014 5;116(1):1–11. 10.1016/j.healthpol.2014.01.007 24485914

[16] Vian T, Kohler JC, Forte G, Dimancesco D. Promoting transparency, accountability, and access through a multi-stakeholder initiative: lessons from the medicines transparency alliance. J Pharm Policy Pract. 2017 6 2;10(1):18. 10.1186/s40545-017-0106-x 28588896 PMC5457587

[17] Moorthy VS, Karam G, Vannice KS, Kieny M-P. Rationale for WHO’s new position calling for prompt reporting and public disclosure of interventional clinical trial results. PLoS Med. 2015 4 14;12(4):e1001819. 10.1371/journal.pmed.1001819 25874642 PMC4396122

[18] Hill SR. Affordable innovation: future directions in pharmaceutical policy. J Pharm Policy Pract. 2015;8 Suppl 1:K1. https://www.ncbi.nlm.nih.gov/entrez/query.fcgi?cmd=Retrieve&db=PubMed&list_uids=25926989&dopt=Abstract 10.1186/2052-3211-8-S1-K125926989

[19] Pepitone K, Sharkey BP. The sun never sets on transparency. Medical Writing. 2016;25(1):15–20. Available from: http://journal.emwa.org/authors-and-authorship/the-sun-never-sets-on-transparency/ [cited 2017 Oct 29].

[20] Siddiqi S, Masud TI, Nishtar S, Peters DH, Sabri B, Bile KM, et al. Framework for assessing governance of the health system in developing countries: gateway to good governance. Health Policy. 2009 4;90(1):13–25. 10.1016/j.healthpol.2008.08.005 18838188

[21] Pyone T, Smith H, van den Broek N. Frameworks to assess health systems governance: a systematic review. Health Policy Plan. 2017 6 1;32(5):710–22. 10.1093/heapol/czx007 28334991 PMC5406767

[22] Baghdadi-Sabeti G, Serhan F. WHO Good Governance for Medicines programme: an innovative approach to prevent corruption in the pharmaceutical sector. Compilation of country case studies and best practices. Geneva: World Health Organization; 2010. Available from: http://www.who.int/healthsystems/topics/financing/healthreport/25GGM.pdf [cited 2017 Dec 7].

[23] Baez-Camargo C, Jacobs E. A framework to assess governance of health systems in low income countries. Working paper series no. 11. Basel: Basel Institute on Governance; 2011. Available from: https://www.baselgovernance.org/publications/385 [cited 2017 Dec 7].

[24] Harmonized NRA assessment tool – prototype I version for comments. Geneva: World Health organization; 2014. Available from: http://www.who.int/immunization_standards/national_regulatory_authorities/tools_revision_2014/en/ [cited 2017 Dec 7].

[25] MDS-3: managing access to medicines and health technologies. Arlington: Management Sciences for Health; 2012. Available from: http://apps.who.int/medicinedocs/en/d/Js19577en/ [cited 2017 Dec 7].

[26] Daniels N. Accountability for reasonableness. BMJ. 2000 11 25;321(7272):1300–1. 10.1136/bmj.321.7272.1300 11090498 PMC1119050

[27] Brinkerhoff DW. Accountability and health systems: toward conceptual clarity and policy relevance. Health Policy Plan. 2004 11;19(6):371–9. 10.1093/heapol/czh052 15459162

[28] Everybody’s business – strengthening health systems to improve health outcomes: WHO’s framework for action. Geneva: World Health Organization; 2007. Available from: http://apps.who.int/iris/handle/10665/43918 [cited 2017 Dec 7].

[29] The Medicines Transparency Alliance. Programmatic review of MeTA phase II [final report]. Geneva: World Health Organization; 2016. Available from: http://apps.who.int/iris/handle/10665/246256 [cited 2017 Oct 7].

[30] Buckland-Merrett GL, Kilkenny C, Reed T. Civil society engagement in multi-stakeholder dialogue: a qualitative study exploring the opinions and perceptions of MeTA members. J Pharm Policy Pract. 2017 1 6;10(1):5. 10.1186/s40545-016-0096-0 28070340 PMC5217617

[31] Vian T, Kohler JC. Medicines Transparency Alliance (MeTA): pathways to transparency, accountability and access. Cross-case analysis and review of phase II. Geneva: World Health Organization; 2016. Available from: http://apps.who.int/medicinedocs/documents/s22502en/s22502en.pdf [cited 2017 Dec 7].

[32] Baghdadi-Sabeti G, Cohen-Kohler J, Wondemagegnehu E. Measuring transparency in the public pharmaceutical sector: assessment instrument. WHO/EMP/MAR/2009.4. Geneva: World Health Organization; 2009. Available from: http://apps.who.int/medicinedocs/documents/s16732e/s16732e.pdf [cited 2016 Jul 10].

[33] Martin J, Ollier L. Evaluation of the Good Governance for Medicines programme (2004–2012): brief summary of findings. WHO/EMP/MPC/2013.1. Geneva: World Health Organization; 2013. Available from: http://apps.who.int/medicinedocs/documents/s20188en/s20188en.pdf[cited 2017 Oct 10].

[34] Good governance in the pharmaceutical sector: report of a World Health Organization technical working group meeting. Tunis, 17−21 March 2014. WHO/EMP/PAU/2014.2. Geneva: World Health Organization; 2014. Available from: http://www.who.int/medicines/areas/governance/ggm_tunis_meeting_report.pdf [cited 2017 Oct 10].

[35] Medicines Transparency Alliance global meeting 2014. Meeting synthesis. WHO/EMP/PAU/2015.1. Geneva: World Health Organization; 2015. Available from: http://apps.who.int/medicinedocs/documents/s21965en/s21965en.pdf[cited 2017 Oct 10].

[36] Report of the bi-regional consultation on good governance for improved access to medicines. Manila, Philippines, 9–11 November 2015. WHO/EMP/PAU/2016.06. Geneva: World Health Organization; 2016. Available from: http://apps.who.int/iris/bitstream/handle/10665/207477/WHO_EMP_PAU_2016.06_eng.pdf?sequence=1&isAllowed=y[cited 2017 Dec 7].

[37] WHO technical working group on good governance in pharmaceutical systems. December 2017. Meeting report. Geneva: World Health Organization; 2018. Available from: http://www.who.int/medicines/areas/governance/en/ [cited 2018 Aug 27].

[38] Fox J. The uncertain relationship between transparency and accountability. Dev Pract. 2007;17(4–5):663–71. 10.1080/09614520701469955

[39] Fox J, Aceron J. Doing accountability differently. A proposal for the vertical integration of civil society monitoring and advocacy. Bergen: U4 Anticorruption Resource Centre, Chr. Michelsen Institute; 2016. Available from: http://www.u4.no/publications/doing-accountability-differently-a-proposal-for-the-vertical-integration-of-civil-society-monitoring-and-advocacy/ [cited 2017 Dec 7].

[40] Mulgan R. “Accountability”: An ever-expanding concept? Public Adm. 2000;78(3):555–73. 10.1111/1467-9299.00218

[41] Bovens M. Analysing and assessing accountability: a conceptual framework. Eur Law J. 2007;13(4):447–68. 10.1111/j.1468-0386.2007.00378.x

[42] Medicines Transparency Alliance Peru. Working together for better access to medicines. WHO/EMP/PAU/2015.7. Geneva: World Health Organization; 2015. Available from: www.who.int/medicines/areas/coordination/country_activity_brochure-Peru.pdf?ua=1http://www.who.int/medicines/areas/coordination/country_activity_brochure-Peru.pdf?ua=1 [cited 2018 Jun 25].

[43] DFID annual review. Medicines Transparency Alliance. June 2013. Geneva: World Health Organization; 2013. Available from: http://www.who.int/medicines/areas/coordination/ar_dfid_2013.pdf?ua=1 [cited 2018 Jun 25].

[44] Making medicines prices transparent in Peru. Geneva: World Health Organization; 2018. Available from: www.who.int/medicines/areas/coordination/medprice_transparent_peru/en/ [cited 2018 Jun 25]

[45] Walkowiak H, Putter S, Strengthening Pharmaceutical Systems (SPS). Pharmaceuticals and the public interest: the importance of good governance. Arlington: Management Sciences for Health; 2011. Available from: http://apps.who.int/medicinedocs/documents/s21019en/s21019en.pdf [cited 2017 Jul 9].

[46] Fair Pricing Forum. Informal advisory group meeting. WHO/EMP/IAU/2017.06. Geneva: World Health Organization; 2017. Available from: http://www.who.int/medicines/access/fair_pricing/fpf_report/en/ [cited 2017 Dec 7].

